# 

**Published:** 1888-06-30

**Authors:** 


					-?"PRICE ONE PENNY.
No. 92} Vol. IV-
June 30th, 1888.
[Registered at the Qeneral Post Office
as a Newspaper.]
Per Annum, 6s. 6d? post free.
Monthly Farts, 6d.; post free, 7}d,
Ditto per Ann., 7s. ed. post free.
jpm^sL?
tern Infirmary
KF
A Weekly Institutional Journal of
cltnct, /Ifcctittim, TKhmng, anb jp>|jtla?tijropg.

				

